# The Conditions for a Path Beyond Capitalism: Comment on "Can a Well-Being Economy Save Us?"

**DOI:** 10.34172/ijhpm.8912

**Published:** 2025-02-03

**Authors:** Lina Brand-Correa

**Affiliations:** Faculty of Environmental and Urban Change, York University, Toronto, ON, Canada.

**Keywords:** Needs, Utilitarianism, Economic Growth, Provisioning Systems, Universal Basic Services

## Abstract

In this commentary I aim to contribute to Ronald Labonté’s recent editorial "Can a Well-Being Economy Save Us?" on the role of well-being economies in providing for everyone’s needs within the limits of our planet. In particular, I add a couple of lessons from the COVID-19 pandemic that can provide the required inspiration to plot a path beyond capitalism – one that is based on hope that change is possible, one where our understanding of well-being is detached from economic growth, one where demand and excess are challenged and one where we go beyond the market to meet our needs.

 In the editorial “Can a Well-Being Economy Save Us?,”^1^ Ronald Labonté uses a historical and public health lens to assess recent advances towards well-being economies worldwide. The author reminds us of the role of activist public health movements and critical public health literature in establishing a critique of market-dominated economic and health systems, mainly through the social determinants of health and planetary health approaches. Labonté also highlights the potential roadblocks ahead of a broader shift towards truly transformational well-being economies. He identifies lack of political traction, risk of performative change and powerful interests as the main barriers for well-being economies.

 Labonté starts with an accurate account of how the COVID-19 pandemic evidenced the failures of our global economic systems, and the limitations of the several “build back better” initiatives that sprung in the aftermath. However, the pandemic left two other important lessons behind. On the one hand, it became proof that rapid and dramatic change is possible, everywhere in the world, as long as there is a sense of urgency at all levels of society. I find this lesson essential in keeping hope that we can still avert the worse effects of climate change, even as the window of opportunity narrows precipitously.

 On the other hand, the pandemic forced us to reflect on what we really need, individually and as a society.^2,3^ The need for well-functioning and coordinated health systems, for affordable and universal access to basic provisioning systems (eg, public health, housing, food, and energy), for social connection and interaction; and it forced us to come up with alternative ways of meeting our needs. Those who were deemed “key workers” worked for those sectors which provide for our needs: health workers, supermarket workers (food), workers in the energy sector, garbage collectors, and teachers. The pandemic forced governments to prioritize, through exemptions and additional support, those areas of the economy which are essential for our well-being, and by implication highlighted those areas that are less important.

 This needs-related lesson is relevant to complement Labonté’s editorial in three ways. First, our understanding of well-being is central to what a well-being economy might look like. Labonté is right in his criticism of welfare economics – around the latter’s tangential regard (at best) for environmental issues and wealth inequalities, its misguided focus on economic growth and its lack of recognition of the importance of common goods – all aspects that well-being economics addresses. However, I would argue that the main difference between welfare and well-being economics is around their underlying understanding of well-being.

 Welfare economics is based on a utilitarian view of well-being. Proposed by Bentham building on the ideas of hedonism, and in the context of a quantitative revolution in economics, utilitarianism attempts to find a measuring unit that quantifies the balance between pleasure and pain.^4^ However, there is no easy way to quantify utility, especially by governments who might want to increase it for their citizens. Therefore, the assumption is that utility is revealed through consumer preferences, who surely make rational consumption choices in a way that maximizes their utility.

 The issues with this type of “Benthamite-subjective-hedonic-individualistic”^5^ view of well-being are not difficult to identify. The most obvious are around the well-documented departures from reality of assumptions of rational behaviour, and around the fact that preferences are unlimited and insatiable (unlike needs). By linking well-being to consumption preferences, the logical consequence for welfare economics is to prioritize economic growth: increases in well-being are obtained through increases in people’s consumption possibilities, which requires increases in economic activity, measured via Gross Domestic Product (which in theory will trickle down to increase everyone’s income).^6^ In contrast, well-being economics understands well-being from a eudaimonic perspective, where needs are universal, incommensurable and satiable, and where cultural specificity and subjectivity comes in the ways in which we satisfy those needs^[1]^.

 Second, as well as highlighting our needs and the most important sectors to keep running, the needs-based lesson from the COVID-19 pandemic highlighted some of those sectors that are less important, perhaps geared towards wants rather than needs. During the pandemic, sectors such as retail and transportation shrank significantly and reductions in economic activity led to significant reductions in CO2 emissions, which have not been seen since. The policy reforms from three well-being reports reviewed by Labonté reflect the usual focus on that which should be kept and improved, but also the less frequent consideration of that which should be eliminated or reduced.

 In other words, most of the policies summarized in Labonté’s Table focus on “lifting floors” – or reducing inequalities and deprivations. This is of course absolutely paramount in any serious attempt to achieve well-being for all. However, and in line with proposals around “sufficiency”^9^ or “sustainable consumption corridors,”^10^ if we are to achieve well-being for all within planetary boundaries, we also need to “lower ceilings,” or reduce certain areas of economic activity – those which do not contribute to the satisfaction of our needs. The three reports propose initiatives to increase and improve certain sectors (such as health and renewable energy). But only the World Health Organization (WHO) Well-Being Framework proposes to reduce fossil fuel extraction and energy consumption. We cannot ignore that demand-side measures are required for avoiding the worse effects of climate change^11^ (eg, limiting luxury consumption), in a similar way that demand-side measures are required to avoid serious health effects (eg, limiting smoking indoors).

 And third, the needs-based lesson from the pandemic also reminded us that we can search for and find alternative ways of satisfying our needs. We can find non-market, non-commodified ways to satisfy our needs. And this is something that Labonté highlights in his editorial when referring to common goods and localized systems of provision. Strengthening the public sector is also prominent in the policy reforms reviewed by Labonté. I would simply like to complement this with the proposals around Universal Basic Services,^12^ which would move the provision of basic needs outside the market, therefore weakening the link between well-being and economic growth (via income and consumption).

 It is worth expanding on this third lesson briefly, because a common counterargument to weakening the link between well-being and economic growth is that many governments rely on tax revenues from ever-expanding economic activity to fund the provision of public services and social care. We can find the answer to such an argument in the work of Buchs and Koch,^13^ who argue that in an economy that works outside the logics of growth, and where the provisioning of basic needs is guaranteed, the demand for many public services would reduce. For instance, it is well known that interventions that tackle social determinants of health (which could be done through Universal Basic Services) result in significant savings in health expenditures down the line, and thus less reliance on economic growth. Here, the role of public health, with a preventative approach, is evident.^14^

 In terms of political traction, it is worth adding to Labonté’s list around the noteworthy actions of the Well-being Economy Governments. Although not directly using the language of well-being economies, the European Parliament’s Beyond Growth Conference in May 2023 discussed many of the issues touched upon here. It was organized by 20 Members of the European Parliament from five different political groups and non-attached.^15^ It was very well attended, with “over 7000 researchers, activists, and politicians gathered in Brussels and online to discuss alternatives to our current economic system.”^16^ One of the main Members of the European Parliament behind it, Philippe Lamberts, reflected on how leading European Union officials are at least engaging in the debate, even if not fully on board.^17^ Is this a sign of political traction or a performative action? That is still to be answered. What is clear is that in order to overcome the influence of powerful opponents to a well-being economy, broad public support is paramount. I am hopeful that the ever-more evident contradictions of a growth-based economy, and its failures to provide well-being for the vast majority of the population, will fuel a bottom-up movement for change. This is especially true for younger generation, who, in their search for secure housing for instance, are keenly aware that the benefits of growth will not trickle down to them.

 I fully agree with Labonté when he concludes “plotting some escape routes out of capitalism is the biggest and most urgent challenge facing efforts to put well-being economics into substantive practice.” And I think insights from the two additional COVID-19 lessons described above might provide the necessary inspiration to plot such routes. A path that merely suggests alternative indicators to Gross Domestic Product is not a path beyond capitalism. It might be a nicer path, but the destination will be as disappointing as our current reality. In my view, paths that go beyond capitalism must build on the hope that change is possible, must clearly detach well-being from economic growth, must not shy away from challenging demand and must encourage non-commodified ways of provisioning for our needs.

## Ethical issues

 Not applicable.

## Conflicts of interest

 Author declares that she has no conflicts of interest.

## Endnotes


^[1]^ The work of Sen, Max-Neef, and Doyal and Gough is central to recent advancements in the understanding of eudaimonic well-being. The work of Sen has sometimes been criticized for its focus on increasing the range of individual life choices (perhaps justifying economic growth for preference satisfaction), however, other authors have highlighted the relational nature of Sen’s approach.^7,8^
